# Supplementary material for: Identification of photoperiod-induced specific miRNAs in the adrenal glands of Sunite sheep (Ovis aries)
Source: Front Vet Sci. 2022 Jul 22;9:888207. doi: 10.3389/fvets.2022.888207 (PMC9354845; doi:10.3389/fvets.2022.888207)
Supplement: Supplementary file 1 [file Data_Sheet_1.ZIP › Supplementary file/miRDeep2 file.zip!/miRDeep2 file/Know_Structure/SPLP42A3/oar-mir-30d.pdf]

miRBase precursor : oar-mir-30d
Total read count : 228154
oar-miR-30d read count : 227551
remaining reads : 603

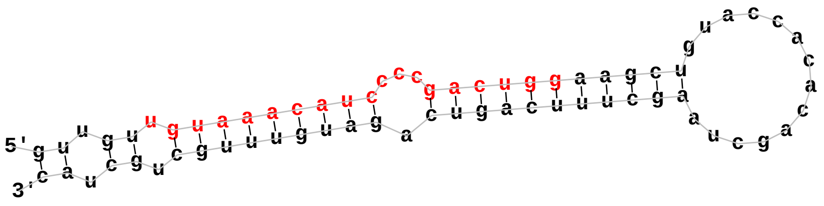

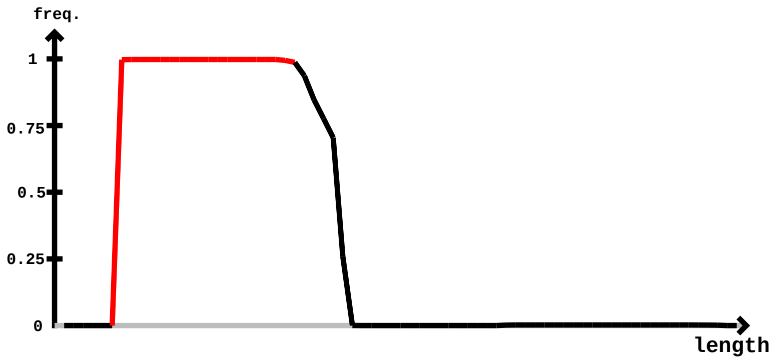


```
       oar-miR-30d
5'- guuguuguaaacauccccgacuggaagcuguaccacacagcuaagcuuucagucagauguuugcugcuac   -3'    exp
    ...((.(((((((((...(((((((((((..............))))))))))).))))))))).))...    reads      mm         sample
    .....uguaaacaucccUgacu................................................    21         1          seq
    .....uguaaacauccccgacu................................................    748        0          seq
    .....uguaaacaucUccgacu................................................    2          1          seq
    .....uguaaacauccccgGcu................................................    1          1          seq
    .....uguaaacaAccccgacu................................................    1          1          seq
    .....uguaaacaAccccgacug...............................................    3          1          seq
    .....uguaaacauccccgacug...............................................    1687       0          seq
    .....uguaaacauccccgacugg..............................................    11449      0          seq
    .....uguaaacauccccgacugga.............................................    20441      0          seq
    .....uguaaacauccccgacuggaa............................................    16163      0          seq
    .....uguaaacauccccgacuggaag...........................................    16331      0          seq
    .....uguaaacauccccgacuggaagc..........................................    101534     0          seq
    .....uguaaacauccccgacuggaagcu.........................................    59011      0          seq
    .....uguaaacauccccgacuggaagcug........................................    56         0          seq
    .....uguaaacauccccgacuggaagcugu.......................................    8          0          seq
    .....uguaaacauccccgacuggaagcuguacc....................................    3          0          seq
    ......guaaacauccccgacug...............................................    1          0          seq
    ......guaaacauccccgacugg..............................................    7          0          seq
    ......guaaacauccccgacugga.............................................    13         0          seq
    ......guaaacauccccgacuggaa............................................    11         0          seq
    ......guaaacauccccgacuggaag...........................................    6          0          seq
    ......guaaacauccccgacuggaagc..........................................    66         0          seq
    ......guaaacauccccgacuggaagcu.........................................    42         0          seq
    .......uaaacauccccgacugga.............................................    1          0          seq
    .......uaaacauccccgacuggaagc..........................................    5          0          seq
    .......uaaacauccccgacuggaagcu.........................................    7          0          seq
    .............................................cuuucagucagauguuugc......    17         0          seq
    .............................................cuuucagucagauguuugcu.....    41         0          seq
    .............................................cuuucagucagauguuugcug....    35         0          seq
    .............................................cuuucagucagauguuugcugc...    61         0          seq
    .............................................cuuucagucagauguuugcugcu..    261        0          seq
    .............................................cuuucagucagauguuugcugcua.    3          0          seq
    ..............................................uuucagucagauguuugc......    1          0          seq
    ..............................................uuucagucagauguuugcu.....    7          0          seq
    ..............................................uuucagucagauguuugcug....    3          0          seq
```

oar-miR-30d

```
guuguuguaaacauccccgacuggaagcuguaccacacagcuaagcuuucagucagauguuugcugcuac
..............................................uuucagucagauguuugcugc...    24    0    seq
..............................................uuucagucagauguuugcugcu..    79    0    seq
..............................................uuucagucagauguuugcugcua.    4     0    seq
```
